# Phytochemical Analysis and Antioxidant Activities of *Prunus africana* Bark, *Leea indica* and *Paullinia pinnata* Leaf Extracts

**DOI:** 10.3390/antiox14060666

**Published:** 2025-05-30

**Authors:** Md Rezaul Karim, Karl E. Miletti-Gonzalez, Alberta N. A. Aryee, Samuel A. Besong

**Affiliations:** 1 Department of Human Ecology (Food and Nutritional Sciences), Delaware State University, Dover, DE 19901, USA; rakrim@desu.edu (M.R.K.); aaryee@desu.edu (A.N.A.A.); 2 Department of Biological Sciences, Delaware State University, Dover, DE 19901, USA; kmiletti@desu.edu

**Keywords:** methanol, phenolics, flavonoids, antioxidants, *Prunus africana*, *Leea indica*, *Paullinia pinnata*

## Abstract

The phytochemical profile and antioxidant activities of *Prunus africana* bark, *Leea indica* and *Paullinia pinnata* leaves from Cameroon were investigated in this study. The yields of pure methanolic extraction were 11.9%, 11.1% and 10.8% in *P. africana* bark, *L. indica* and *P. pinnata* leaves, respectively. The total phenolic content was 189.0 ± 16.93, 163.6 ± 14.73 and 114.6 ± 10.38 mg GAE/g and total flavonoid content was 43.25 ± 6.43, 28.31 ± 4.44, and 19.75 ± 4.03 mg RU/g in *P. africana* bark, *L. indica* and *P. pinnata* leaves, respectively. The antioxidant activities of the plants were evaluated by DPPH, ABTS and FRAP assays. The IC_50_ evaluated in *P. africana* bark, *L. indica* and *P. pinnata* leaves was 109.5 ± 13.2, 132.1 ± 18.7 and 156.1 ± 21.9 µg/mL for DPPH and 98.1 ± 4.8, 101.3 ± 12.1 and 133.9 ± 16.0 µg /mL for ABTS assay. The FRAP value was 61.1 ± 1.5, 50.5 ± 1.5 and 43.4 ± 2.1 µMFe^2+^/g in the same sequence. The functional groups for the corresponding phytochemicals, including alkane, alkene, aliphatic ether, ester, amine, α, β-unsaturated ester, alcohol, phenol, carboxylic acid, and aliphatic ketone, were identified through fourier-transform infrared analysis. The identified and quantified phenolic acids in this study were methyl-4-hydroxybenzoic, caffeic, protocatechuic and p-coumaric acid, identified using high-performance liquid chromatography.

## 1. Introduction

Bioactive substances found in plants and herbs have been effectively employed to treat metabolic disorders and halt the progression of chronic illnesses [1]. It has been well documented that people have been using various herbal plants for the prevention and improvement of various diseases over the past 60,000 years [2]. Nowadays, herbal remedies are being chosen as an alternative medication among 80% of the populations in the world because of the claim that they have fewer side effects compared to conventional drugs [3,4]. Based on their role in plant metabolism, phytochemicals are mainly categorized into primary and secondary metabolites [5]. Primary metabolites protect plants against insects as well as herbivores, and secondary metabolites are broadly used for therapeutic purposes [6]. Secondary metabolites are reported to function against carcinogenesis, inflammation, diabetes, oxidation, allergy and microbial infection [7,8,9]. Phytochemicals neutralize reactive oxygen species (ROS) levels, these being mostly responsible for the onset and development of various complex diseases like cancers [10]. These reactive oxygen species or free radicals, due to having unpaired electrons, are extremely unstable and seek to stabilize themselves by either donating or accepting an electron or hydrogen atom from other molecules [11]. These free radicals are naturally synthesized in the body during various metabolic processes or by external sources like UV radiation, pollution, tobacco smoke, and certain chemicals that initiate many complex diseases [12]. Phytochemicals have antioxidant activities that can neutralize free radicals by donating or accepting electrons and hydrogen atoms [13]. So, phytochemicals play an important role in reducing the risk of various diseases like cancer, asthma, diabetes mellitus, neurodegenerative diseases, cardiovascular diseases and infections, based on the ability to neutralize reactive oxygen species/free radicals [14]. Consuming an antioxidant-rich diet or using antioxidant supplements may help to mitigate the harmful effects of free radicals and reduce the risk of associated health problems [15].

*Prunus africana* (*P. africana*), also known as African cherry, is a type of evergreen tree indigenous to sub-Saharan Africa’s highland regions [16]. Mature stem grows up to 1 m in diameter and approximately more than 40 m in height [17]. It was reported in a previous study that this plant possesses therapeutic activities due to containing bioactive phytochemicals [18]. Various parts of *P. africana* are typically used in lowering stomach pain, wound dressing, arrow poison, fevers, gonorrhea, malaria and kidney disease [19]. According to traditional African healers, *P. africana* extract is the most powerful and successful treatment for prostate cancer, particularly in Cameroon [20,21].

*Leea indica (L. indica),* also known as bandicoot berry, is an evergreen perennial shrub or short tree that grows up to 2–16 m in height. It has alternating, stalked, twice- or triple-pinnate leaves that are roughly 45 to 60 cm long. *L. indica* is widely grown in the forests of tropical and subtropical regions like India, Bangladesh, China, Bhutan, and Malaysia [22]. It was reported in previous studies that this plant has potency against inflammation, carcinogenesis, diabetes, microbial infection, cytotoxicity, oxidation, enzyme inhibition, analgesia, hypoglycemia, hypolipidemia, and diarrhea [23,24,25,26,27,28]. *L. indica* leaves were also reported to have several potential bioactive phytochemicals [29,30].

*Paullinia pinnata* Linn. (*P. pinnata*) is a climbing shrub holding green leaves with multiple ovate, elliptic or rhomboid leaflets, and is available in South American and African countries [31]. This plant is commonly used as traditional medicine for treating bacterial infection, menstrual pain, erectile dysfunction, sores, snake bites, dysentery, coughs, rheumatoid arthritis, wounds, and malaria in Nigeria, Ghana and Togo [32,33,34]. Numerous bioactive phytochemicals have also been reported in this plant [35].

Based on the beneficial health claims of the above three plants, it is important to study their phytochemical analysis and identify more unknown potent phytoconstituents. The present study aimed to investigate the phytochemical constituents and antioxidant potentials, and explore new phenolic compounds of these plants. This study will reveal the chemical basis in support of antioxidant activities of these plants and uncover the way forward for further research to validate conventional therapeutic claims. The findings of this study will help to assess the nutritional and medicinal values of these plants as well.

## 2. Materials and Methods

### 2.1. Plant Materials and Chemicals

*P. africana* bark, *L. indica* leaves and *P. pinnata* leaves were harvested, air dried and packaged in Cameroon, then transported to the Food Chemistry lab at Delaware State University, USA. Sigma Aldrich in St. Louis, MO, USA, and Thermo Fisher Scientific in Waltham, MA, USA, were the suppliers of the solvents and reagents.

### 2.2. Extract Preparation

The plant parts were pulverized through a coffee grinder and stored at 4 °C until analysis. Twenty-five gm of the powdered plant samples were placed in a sterile dry 600 mL glass conical flask and dissolved with 200 mL of 100% methanol and continuously stirred at room temperature for 24 h. They were then filtered using Whatman’s filter paper No.1. The obtained broth was transferred into another sterile dry 500 mL glass conical flask using a piece of clean cotton wool to sieve out any powdered debris. The filtrate was then concentrated using a rotary evaporator (Buchi R-300 Rotavapor Rotary, New Castle, DE, USA) at 40 °C. All the extracts were kept in the refrigerator at 4 °C for further analysis.

### 2.3. Qualitative Screening of Phytochemicals

Qualitative analysis of the phytochemicals was screened following the methods below.

#### 2.3.1. Detection of Alkaloids

An amount of 0.1 gm of each plant extract was added to 5 mL of 1% H_2_SO_4_ in a steam bath. The solution was filtered and mixed with the following reagents. The colored precipitation indicated the presence of alkaloids.

##### Mayer’s Test

The filtrate was taken in separate test tubes and then 1 mL of Mayer’s reagent was added dropwise by the side of the test tube. The formation of greenish colored or cream precipitate indicated the presence of alkaloids [36].

##### Wagner’s Test

The filtrate was placed in different test tubes and then 1 mL of Wagner’s reagent was added dropwise by the side of test tube. The formation of reddish-brown precipitate indicated the presence of alkaloids [36].

#### 2.3.2. Detection of Phenolics

##### Ferric Chloride Test

An amount of 2 mL of each extract was added in different test tubes followed by adding a few drops of FeCl_3_ solution. The formation of a greenish-black color indicated the presence of phenolic compounds [37].

#### 2.3.3. Detection of Flavonoids

##### Sodium Hydroxide Test

An amount of 2 mL of each extract was placed in separate test tubes, followed by adding 2 mL of 10% NaOH solution. After the addition of NaOH, a yellow color was observed; however, adding 70% HCl caused the yellow color to disappear, indicating the presence of flavonoids [36].

#### 2.3.4. Detection of Terpenoids

An amount of 2 mL of each extract was placed in different test tubes and then 2 mL of chloroform was added. The test tubes were shaken vigorously and then filtered. To the filtrate, 2 mL of conc. H_2_SO_4_ was added by the side of the test tube. After the addition of H_2_SO_4_, a reddish-brown steroidal ring was observed at the inter phase, indicating the presence of terpenoids [38].

#### 2.3.5. Detection of Tannins

##### Ferric Chloride Test

An amount of 2 mL of each extract was mixed with 5 mL of water in different test tubes, and was heated and then filtered; 2 mL of the filtrate was taken in another test tube, and a few drops of FeCl_3_ solution were added to the test tube. The formation of a blue or greenish-black color that turned to olive green as more FeCl_3_ was added indicated the presence of tannins [36].

#### 2.3.6. Detection of Saponins

##### Froth Test

An amount of 2 mL of each extract was taken in different test tubes and 10 mL of distilled water was added. All the test tubes were vigorously shaken for 1 min and left to stand for 30 min. The formation of foam on the top edge of the reaction mixture indicated the presence of saponins [37].

### 2.4. Quantitative Analysis of Phytochemicals

#### 2.4.1. Determination of Percentage of Yield

The percentage of yield of the extracts was measured by using the following formula.

% of yield = (Weight of dry extract obtained in g/Weight of powder sample in g) × 100

#### 2.4.2. Determination of Total Phenolic Content (TPC)

The TPC of the extracts was measured following this procedure [39]. Five mg of each extract was dissolved in one ml of 60:40 (*v*/*v*) methanol: water buffer solution; 100 µL of the extract solution (sample) and 100 µL of buffer solution (blank) were placed in separate wells of a 96-well plate. Two ml of 2% Na_2_CO_3_ solution was added in both wells, mixed gently and kept at room temperature for two minutes. Then, 100 µL of 50% Folin–Ciocalteu reagent was added to all the wells and kept in incubation at room temperature for thirty minutes. The absorbance of the samples at different concentrations of gallic acid standard in the range of 0.05–0.11 mg/mL was measured at 750 nm using a Synergy HTX multi-mode reader (Bio Tek, Winooski, VT, USA). The concentration of TPC in the extracts was calculated from the standard curve of gallic acid and expressed as milligrams of gallic acid equivalent per gram of extract (mg GAE/g). This was conducted in triplicate.

#### 2.4.3. Determination of Total Flavonoid Content (TFC)

TFC was determined following the Stankovic method [40]. Four mg of each extract was dissolved in one ml of 100% methanol. Then, 200 µL of the extract solution (sample) and 200 µL of methanol (blank) were placed in separate wells of a 96-well plate. One ml of 2% AlCl_3_ was added to both wells, mixed gently and incubated at room temperature for one hour. The absorbance of the samples with different concentrations of rutin standard in the range of 0.005–0.03 mg/mL was measured at 415 nm using a Synergy HTX multi-mode reader (Bio Tek, Winooski, VT, USA). The concentration of total flavonoid content was measured from the standard curve of rutin and expressed as mg rutin per gram of extract (mg RU/g). This was conducted in triplicate.

### 2.5. Antioxidant Activity Assays

The antioxidant activity of a sample could be estimated through several types of assays [41]. Tests on the % of 2,2-diphenyl-1-picrylhydrazyl (DPPH) and % of (2,2-azino-bis (3-ethyl-benzothiazoline-6-sulfonic acid)) (ABTS) radical scavenging activity, ferric ion reducing antioxidant power (FRAP), and inhibition concentration (IC_50_) assays were carried out in this study, as these are more reliable and widely used. The methods are described below.

#### 2.5.1. DPPH Radical Scavenging Activity

The DPPH radical scavenging activity was determined following this method [42]: 50 µL of 5 mg/mL sample extract solution was added to 150 µL of 100% methanol in a well of a 96-well plate and mixed gently; 50 µL of 0.12 mg/mL of DPPH in methanol was added and the mixture was kept in incubation at room temperature for thirty minutes in the dark. After making a blank with a solution made up of 150 µL methanol and 50 µL test solution, the absorbance of the samples and the control (150 µL methanol and 50 µL DPPH) were taken at 515 nm using a Synergy HTX multi-mode reader (Bio Tek, Winooski, VT, USA) in dim lighting. The degree of DPPH decolorization from purple to yellow indicated the scavenging capacity of the extract. Lower absorbance indicated higher free radical-scavenging activity. The % of DPPH radical scavenging activity was calculated following the following formula: % of DPPH scavenging = [(Absorbance of control − Absorbance of sample)/Absorbance of control] × 100. This assay was conducted in triplicate.

#### 2.5.2. ABTS Radical Scavenging Activity

ABTS radical scavenging activity was determined using the following method, with slight modifications [43]. The ABTS working solution was prepared by combining 7 mM ABTS solution and 2.45 mM potassium persulfate (K_2_S_2_O_8_) at a ratio of 1:1 (*v*/*v*). The ABTS solution was diluted with ethanol to adjust the absorption value to 0.70 ± 0.02 at 734 nm. Then, 200 µL of the ABTS working solution was mixed with 10 µL of the sample in a 96-well plate. Then, the mixture was left for 10 min in the dark and the absorbance was measured at 734 nm. The ABTS radical scavenging activity was calculated using the following formula: ABTS radical scavenging rate (%) = [(Absorbance of control − Absorbance of sample)/Absorbance of control] × 100. This assay was conducted in triplicate.

#### 2.5.3. Determination of IC_50_ and Anti-Radical Power (ARP)

IC_50_ is the concentration of an antioxidant that can reduce the initial absorbance of DPPH and ABTS radicals by 50% [44]. It was measured by plotting the % of DPPH and ABTS radical scavenging activity versus the sample extract concentration [45]. The lower the IC_50_ value, the higher the antioxidant power of the sample extract [46]. The ARP of an antioxidant indicates its antioxidant potential [44]. ARP was calculated as the reciprocal value of the inhibitory concentration (IC_50_) [47]. The higher the ARP value, the higher the free radical scavenging activity. This assay was conducted in triplicate.

#### 2.5.4. FRAP Assay

The FRAP assay was carried out in line with the following procedure, with slight modification [48]. Two mg of each extract was dissolved in one ml of methanol. The FRAP working reagent was made by mixing together 300 mM acetate buffer at pH 3.6 with 10 mM TPTZ and 20 mM ferric chloride solution (FeCl_3_.6H_2_O) at a ratio of 10:1:1 (*v*/*v*/*v*) just before use. A 0.1 mL aliquot of sample extract solution was added to three ml of FRAP working reagent in a 96-well plate and mixed carefully. In addition, 1 mM of ferrous sulfate (FeSO_4_.7H_2_O) standard concentration (range of 5–100 µM) was also placed in a 96-well plate and was incubated in a water bath (BÜCHI, New Castle, DE, USA) at 37 °C for four mins. The absorbance of the samples and standard concentration was measured at 593 nm using a Synergy HTX multi-mode reader (Bio Tek, Winooski, VT, USA). The FRAP value was calculated from the standard curve of FeSO_4_.7H_2_O and expressed as µM Fe^2+^ per gram of extract. This assay was conducted in triplicate.

### 2.6. Fourier-Transform Infrared (FTIR) Spectroscopy

FTIR was used to identify the functional groups of the phytochemicals existing in the sample extracts, comparing the absorption bands of the sample spectrum with those of the standard spectra library. The results of FTIR analysis indicate the presence of various functional groups. In total, 10 mg of each dried extract was loaded on an FTIR spectroscope (Shimadzu, Kyoto, Japan) at room temperature with a scan range from 400 cm^−1^ to 4000 cm^−1^.

### 2.7. HPLC Analysis

HPLC analysis of the sample extracts was carried out using a Shimadzu LC-20AB Prominence chromatograph equipped with a Shimadzu SPD-M20A PDA detector (Kyoto, Kyoto prefecture, Japan). Separation of the compounds was performed through C18 reversed-phase Phenomenex LC column (5 µm × 250 mm × 4.6 mm) (Phenomenex, Torrance, CA, USA) at a fixed temperature of 25 °C. An amount of 10 µL of the sample extract solution was injected at a mobile phase of 2.5% acetic acid in HPLC-grade water (*v*/*v*) (solvent A) and 100% acetonitrile (solvent B). Gradient elution followed, and the flow was at a fixed rate at 1 mL/min. The mobile phase was as follows: 0.0–0.01 min, 0–3% B; 0.01–10 min, 3–10% B; 10–12 min, 10–15% B; 12–64 min, 15–24% B; 64–69 min, 24–50% B; 69–74 min, 50–95% B; 74–75 min, 50–95% B; the total run time was 75 min. Seven phenolic acids—trans-sinapic, methyl–4-OH-benzoic, protocatechuic, vanillic, trans-ferulic, p-coumaric and caffeic acid—were used as external standards, and syringic acid was used as an internal standard. The sample extracts and internal and external standards were prepared in 100% HPLC grade methanol at 10 mg/mL as stock solutions. However, a concentration of 1 mg/mL for both the standards and 9 mg/mL for the sample extracts were applied for HPLC analysis. Peaks of the sample chromatograms for phenolic acids were identified by comparing the retention times with those of the internal and external standards at 250 nm. The identified phenolic acids were quantified using relative response factors followed by the method [49].

#### 2.7.1. Calculation of Relative Response Factors (RRFs)

External and internal standard solutions were mixed at a ratio of 1:1 (*v*/*v*), then subjected to HPLC analysis. The peak areas, obtained from the above analysis, were applied to the following equation for RRF calculation.

RRF = (A*_ES_*/W*_ES_*) ÷ (A*_IS_*/W*_IS_*)

where A*_ES_* and A*_IS_* indicate the peak area and W*_ES_* and W*_IS_* are the weight in injection volume of external and internal standards, respectively, applied in HPLC analysis.

#### 2.7.2. Quantification of Phenolic Acids

Quantity of phenolic acids in the extract = (A*_Phenolics_*÷A*_IS_*) × (W*_IS_* ÷ RRF) × (1 ÷ W*_sample_*)

where A*_Phenolics_* and A*_IS_* are the peak area of respective phenolics and internal standard in sample chromatogram. W*_IS_* and W*_sample_*are the weight in injection volume of the internal standard and sample used in HPLC analysis.

### 2.8. Statistical Analysis

Data were analyzed using GraphPad Prism, version 10 (GraphPad Software Corp, San Diego, CA, USA). Values were expressed as the mean of three determinants (*n* = 3) ± SD, and a one-way ANOVA with error bars representing differences in standard deviation was determined using Tukey’s multiple comparisons tests.

## 3. Results and Discussion

### 3.1. Phytochemical Screening

All the extracts were screened qualitatively to detect the presence or absence of various bioactive compounds. Alkaloids, phenolics, flavonoids, tannins, and terpenoids were notably detected in all the extracts, while saponins were absent in *L. indica* and *P. pinnata* leaves. The results are shown in Table 1.

### 3.2. Percentage of Yield

The percentage of yield for *P. africana* bark extract was 11.9%, which was in-between the results (24.45% and 8.13%) from the studies in *P. africana* leaves [50,51]. The result in *L. indica* leaves in this study was 11.1%, which was slightly higher than those in a previous study (5.3–7.81%) using different solvents [52]. The percentage of yield for *P. pinnata* leaves in this study was 10.8%, which is slightly higher than those from a study (8.31% and 8%) in ethanol and aqueous methanol extracts [31,53].

### 3.3. Total Phenolic and Flavonoid Content (TPC and TFC)

**Figure 1:**
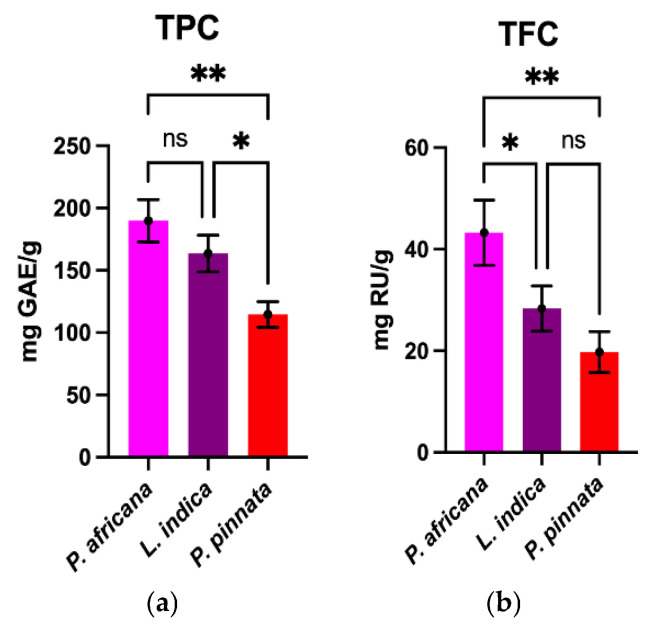
Comparison of TPC and TFC among plant extracts. Results are expressed as mean ± SD (*n* = 3). One-way ANOVA with error bars representing standard deviation differences, where * *p* < 0.05 and ** *p* < 0.01 were considered as significant; ns: not significant.

TPC and TFC determined in the sample extracts are graphically shown in Figure 1. The TPC value obtained in this study in *P.africana* bark was 189.0 ± 16.93 mg GAE/g, which was higher than the reports in previous studies (12.20 to 69.2 mg GAE/g) [54,55,56]. The TPC value of *L. indica* leaves in this study was 163.6 ± 14.73 mg GAE/g, which is in-between the results (65.20–240.0 mg GAE/g) in previous studies [52,57]. However, the TPC value of *P. pinata* leaves obtained in this study was 114.6 ± 10.38 mg GAE/g, which is lower than the results (150 mg GAE/g) in a previous study [53], but higher than the 33.4 mg tannic acid equivalent/g reported in a different study [33]. The findings obtained in this study demonstrate that *P. africana* bark contained the highest TPC, followed by *L. indica* and *P. pinnata* leaves.

The TFC obtained in *P. africana* bark was 43.25 ± 6.43 mg rutin/g, which is close to the value (36.4 mg RU/g) reported in a previous study in *P. africana* [56]. The TFC of *L. indica* leaves was 28.31 ± 4.44 mg rutin/g, which is lower than the result (1.95 ± 0.02 g quercetin/g) in a previous study [57]. However, the TFC obtained for *P. pinnata* was 19.75 ± 4.03 mg rutin/g, which is higher than the results (4.3 mg of quercetin equivalents/g) from a similar study [33]. So, the result from this study showed that *P. africana* bark has the highest TFC, followed by *L. indica* and *P. pinnata* leaves. In all the plant extracts in this study, TPC was found to be higher than TFC, because most flavonoids are in fact phenolics as well [58]. The TPC and TFC values obtained in this study were somewhat different in comparison with the previous studies due to differences in methodologies, reference standard, extraction methods, sample-to-solvent ratios, harvesting time, geographical location, environmental conditions and the sample size of plant materials [55,56,59,60].

### 3.4. Antioxidant Activities

**Figure 2:**
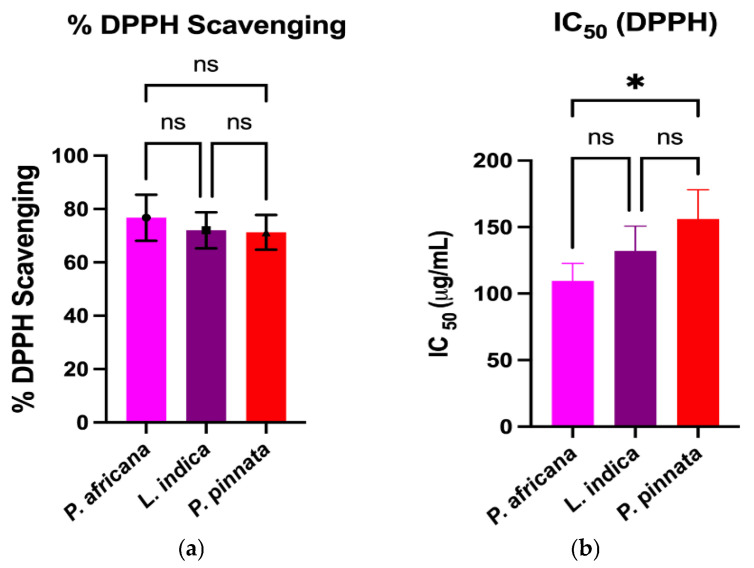

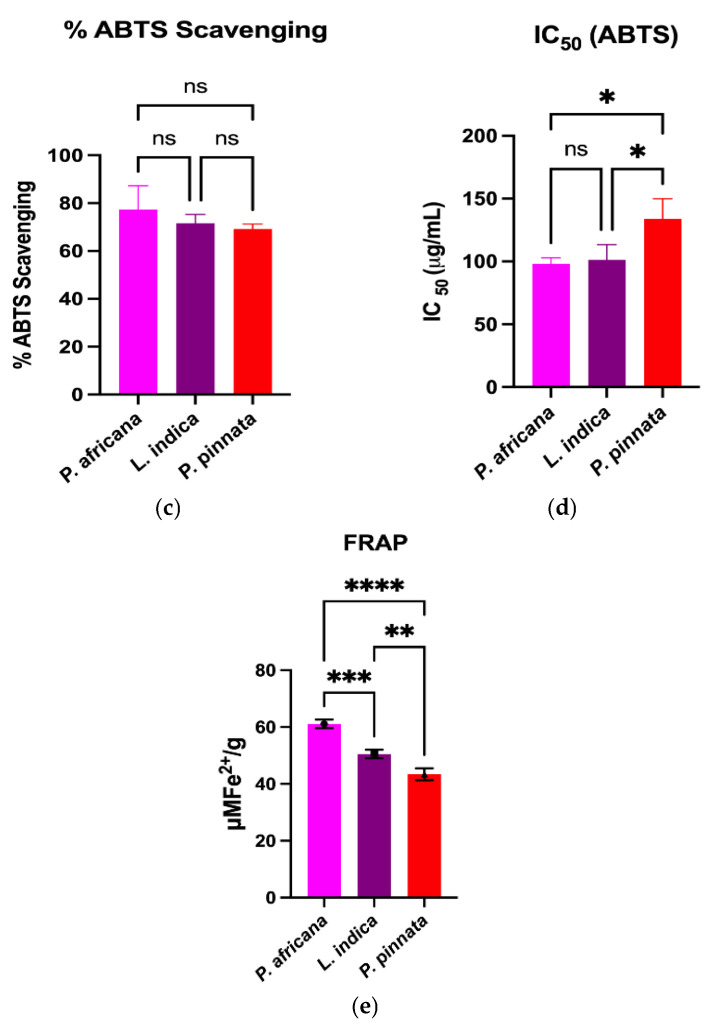
Comparison of antioxidant activities among plant extracts. Results are expressed as mean ± SD (*n* = 3). One-way ANOVA with error bars representing standard deviation differences, where * *p* < 0.05, ** *p* < 0.01, *** *p* < 0.001, and **** *p* < 0.0001 were considered as significant; ns: not significant.

The DPPH and ABTS assays measure the capacity of a hydrogen atom or a single electron donation of an antioxidant to scavenge the stable radical DPPH and ABTS [61]. Results and comparison of antioxidant activities in the extracts are graphically shown in Figure 2. All the extracts exhibited potent antioxidant activities through the percentage of DPPH and ABTS radical scavenging, IC_50_ and FRAP assays. In this study, the *P. africana* bark extract showed the *%* of DPPH and % of ABTS radical scavenging of 76.76 ± 8.62% and 77.4 ± 9.9%, with IC_50_ values of 109.5 ± 13.2 µg/mL and 98.1 ± 4.8 µg/mL, and ARP values of 0.009 µg/mL and 0.01 µg/mL. In a previous study, the IC_50_ of DPPH was 0.036 mg/ mL in *P. africana* bark extract [62].

The % of DPPH and % of ABTS radical scavenging activity in *L.indica* leaf extract were 72.06 ± 6.73% and 71.6 ± 3.7%, with IC_50_ values of 132.1 ± 18.6 µg/mL and 101.3 ± 12.1 µg/mL, and ARP values of 0.007 µg/mL and 0.009 µg/mL, respectively, which is close to a previous report of a % DPPH scavenging value of 80.98 ± 0.42% and an IC_50_ of 0.0398 ± 0.01 mg/mL [57]. However, *P. pinnata* leaf extract showed the lowest activity of 71.28 ± 2.01% and 69.24 ± 2.02% in DPPH and ABTS radical scavenging activity, with IC_50_ values of 156.1 ± 21.9 µg/mL and 133.9 ± 16.0 µg/mL, and ARP values of 0.006 µg/mL and 0.007 µg/mL, which is lower than a similar report of a *%* DPPH radical scavenging value of 96.0% and an IC_50_ of 0.04 mg/mL [33]. The FRAP of *P. africana* bark, *L. indica* and *P. pinnata* leaves in this study were 61.1 ± 1.52, 50.47 ± 1.51, and 43.37 ± 2.1 µMFe^2+^/g, respectively. The values found in this study were lower than the previous results [63,64], higher than the findings [65] and close to the values [66]. The result indicates that *P. africana* bark extract has the highest FRAP activity, followed by *L. indica* and *P. pinnata* leaves. The antioxidant activity of any plant extract is typically considered as very powerful when IC_50_ < 50 μg/mL, strong for an IC_50_ = 50–100 μg/mL, moderate for IC_50_ = 101–250 μg/mL, weak for IC_50_ = 250–500 μg/mL and inactive for when IC_50_ > 500 μg/mL [67,68]. According to the findings in our study, *P. africana* bark extract showed strong and moderate antioxidant activity based on the % of ABTS and % of the DPPH assay. However, *L. indica* and *P. pinnata* leaf extracts showed moderate antioxidant activity in this study.

### 3.5. FTIR Spectra of the Sample Extracts

**Figure 3:**
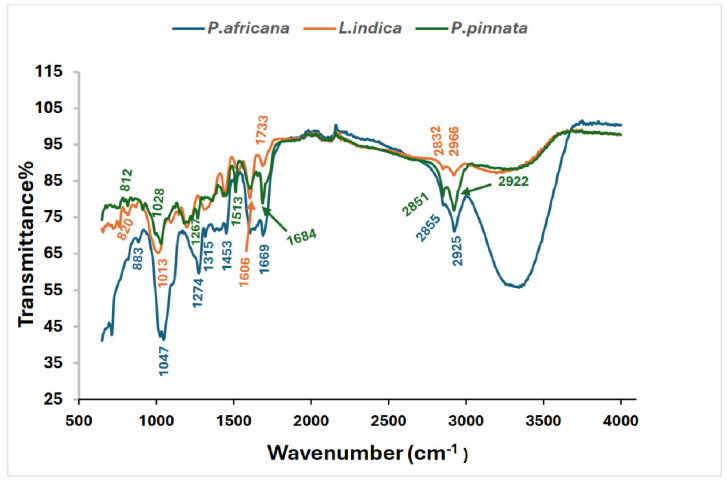
FTIR Spectrum of *P. africana* bark, *L. indica* and *P. pinnata* leaves.

All the potential bands labeled with wave numbers and possible functional groups are shown in Table 2 and Figure 3. *P. africana* bark extract showed the characteristic absorption bands at 887, 1025, 1047, 1274, 1315, 1513, 1688, 2855, and 2925 cm^−1^, representing C=C, C–N/F C–O, C–H, C=O, C–H, N–H, and O–H functional groups, with the corresponding possible bioactive phytochemicals shown in Table 2. Similar functional groups were reported in *P. africana* bark extract in a previous study [62]. In the extract of *L. indica* leaves, the presence of C=C, C–N/F C–O, C–H, C=O, C–H, N–H, and O–H functional groups found in this study was comparable with those reported in a previous study [69]. The absorption bands at 820, 1028, 1241, 1375, 1438, 1513, 1606, 1990, 2851, and 2922 cm^−1^ detected in *P. pinnata* leaf extract, representing the corresponding functional groups, were also closer to the results for *P. pinnata* leaves in a previous study [31].

### 3.6. Identified and Quantified Phenolic Compounds in the Sample Extracts

**Figure 4:**
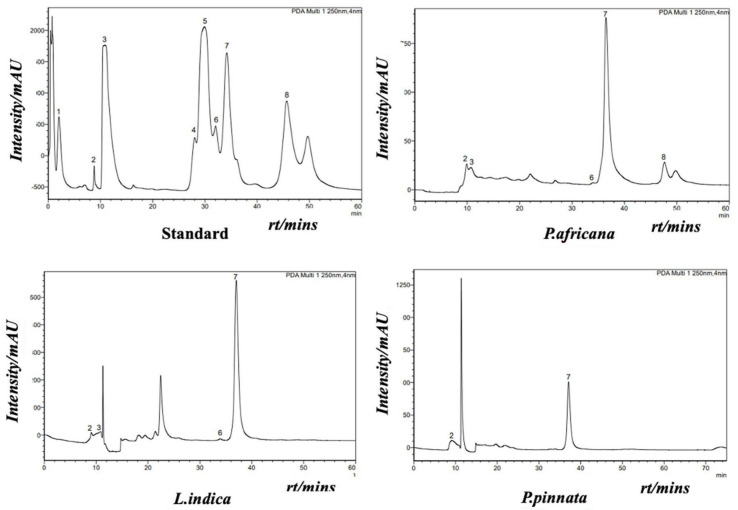
HPLC chromatogram of internal/external standard mix (**upper left**): 1: trans-sinapic; 2: methyl-4-hydroxy-benzoic; 3: protocatechuic; 4: vanillic; 5: trans-ferulic acid; 6: P-coumaric; 7: syringic (internal standard); and 8: caffeic acid. HPLC chromatograms of *P. africana* bark (**upper right**), *L. indica* leaves. (**below left**) and *P. pinnata* leaves (**below right**).

**Figure 5:**
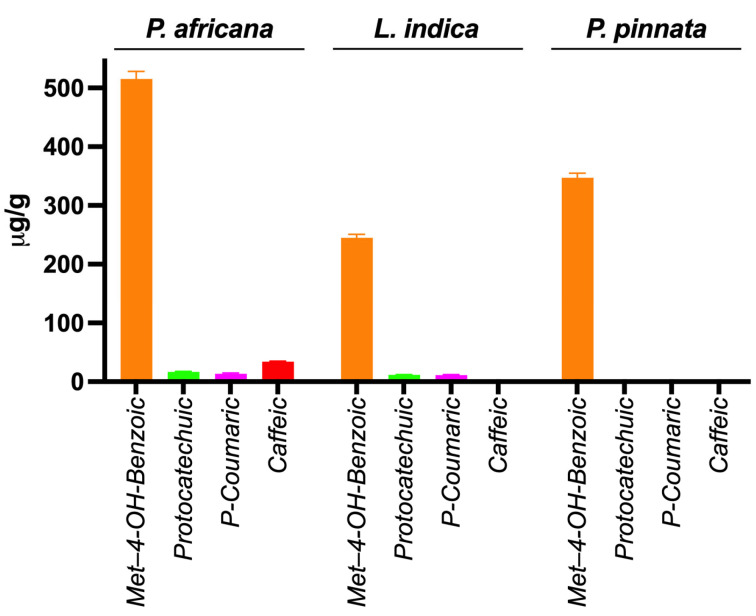
Graphical comparison of phenolic acids in extracts.

Phenolic compounds in plants are identified by one or more aromatic rings and several attached hydroxyl groups. Extract of *P. africana* bark, *L. indica and P. pinnata* leaves were found to have four, three and one phenolic acid, respectively, in this study. All the chromatograms are shown in Figure 4, the quantity of phenolic acids is shown in Table 3 and a graphical comparison is shown in Figure 5. Methyl-4-hydroxybenzoic acid was the common phenolic acid in all the samples investigated. In this study, *P. africana* bark showed the highest methyl-4-hydroxybenzoic acid (515.54 ± 12.75 µg/gm), followed by *P. pinnata* (347.4 ± 7.77 µg/gm) and *L. indica* (244.8 ± 6.04 µg/gm), respectively. In a similar study, 324 μg/g of 4-OH-benzoic acid was reported in ethanolic extract of callus cultures of Lavandula angustifolia [70]. Protocatechuic acid values obtained in this study were 16.78 ± 0.64 and 11.56 ± 0.5 µg/gm in *P. africana* and *L. indica*, respectively; 20.8–44.7 µg/gm of the same phenolic acid was found in ethanolic extract of *P. spinosa* L. fruits at a different temperature [71]. Protocatechuic acid shows numerous health-promoting activities due to its anti-oxidative, anti-inflammatory, anti-diabetic, anti-carcinogenic, cardiovascular and neuroprotective potentials [72]. P-coumaric acid values of 13.38 ± 1.51 µg/gm and 11.33 ± 1.04 µg/gm were obtained in *P. africana* bark and *L. indica* leaves, which was less than the amount 64.0 ± 3.0 µg/gm found in methanolic extract of *Zanthoxylum acanthopodium* leaves [73]. This phenolic acid shows hepatoprotective activity by suppressing the apoptotic gene protein caspase-3, gastroprotective activity, and anti-inflammatory and immunomodulatory effects [74,75,76]. Caffeic acid (34.26 ± 0.61 µg/gm) was found only in *P. africana* bark, which was a similar value to that of 34.0 ± 2.0 µg/gm found in methanolic extract of *Zanthoxylum acanthopodium* leaves [73]. Caffeic acid has potential activities including anti-diabetic, anti-atherosclerotic, anti-obesity, anti-cancer, antiviral, antibacterial and neuroprotective [77]. It has been reported that the phenolics and flavonoids of plants mainly play the role of their pharmacological efficacy [78,79]. So, the plants investigated in this study showed their potent antioxidant activities due to having these phenolic compounds. 

## 4. Conclusions

Medicinal plants are a vital source of important phytoconstituents and are being widely used in industries for the development of drugs and functional foods. The plants investigated in this study have been shown to have a good source of alkaloids, flavonoids, terpenoids, saponins, tannins, and phenolics, which play a crucial role in their potent antioxidant activities. This study has demonstrated that these plants contain a significant amount of phenolic and flavonoid compounds. From the antioxidant activity assays, we found that *P. africana* bark extract possesses strong antioxidant activities and *L. indica* and *P. pinnata* leaf extracts contain moderate antioxidant activities. The obtained phenolic compounds in this study play a crucial role in their antioxidant activities. The presence of the phytoconstituents in these plants validates their alternative therapy claims. However, this needs to be studied more to understand the precise health benefits of these plants for their rationale utilization in food and pharmaceutical industries.

## Figures and Tables

**Table 1:** Phytochemical screening of the extracts.

Plant Name	Alkaloid	Flavonoid	Terpenoids	Saponins	Tannins	Phenol	Steroids
*P.africana* bark	++	+++	++	+	++	+++	++
*L. indica* leaves	++	++	+	−	++	++	++
*P. pinnata* leaves	+	++	+	−	+	++	+

“+”, “++”, “+++”, and “−” denote a presence, high presence, an extremely high presence and an absence, respectively.

**Table 2:** FTIR spectral wave numbers and functional groups obtained in extracts.

	Wavenumber (cm^−1^)		Band No.	Band Assignments	Possible Compounds
	Band Range (Literature) (cm^−1^)	Band Range (Experimental)			
*P. africana* bark	1000–650	887	1	C=C	alkene
	1400–1000	1025, 1047, 1274, 1315	2, 3, 4, 5, 6	C–N/F, C–O	amine, fluoro compound, alcohol, aliphatic ether, ester, phenol.
	1600–1300	1513	7	C–H	alkanes
	2000–1650	1688	8	C=O	carboxylic acid, aliphatic ketone; α, β-unsaturated ester.
	4000–2500	2855, 2925	9, 10, 11	C–H, N–H, and O–H	alkane, alkene, amine salt, and alcohol, carboxylic acid.
*L. indica* leaves	1000–650	764, 868	2	C=C	alkene
	1400–1000	1013, 1095, 1144, 1196, 1312	3, 4, 5, 6, 7	C–N/F, C–O	amine, fluoro compound, alcohol, aliphatic ether, ester, phenol.
	1600–1300	1442, 1531	8, 9	C–H	alkanes
	2000–1600	1602, 1684	10, 11	C=O	carboxylic acid, aliphatic ketone; α, β-unsaturated ester.
	4000–2500	2851, 2922	12, 13	C–H, N–H, and O–H	alkane, alkene, amine salt, and alcohol, carboxylic acid.
*P. pinnata* leaves	1000–650	820	1	C=C	alkene
	1400–1000	1028, 1241, 1375	2, 3, 4	C–N/F, C–O	amine, fluoro compound, alcohol, aliphatic ether, ester, phenol.
	1600–1300	1438, 1513	5, 6	C–H	alkanes
	2000–1600	1606, 1990	7, 8	C=O	carboxylic acid, aliphatic ketone; α, β-unsaturated ester.
	4000–2500	2851, 2922	9, 10	C–H, N–H, and O–H	alkane, alkene, amine salt, and alcohol, carboxylic acid.

**Table 3:** Quantity of phenolic acid in the extracts.

Plant Name	Phenolic Acids (µg/gm of Extract)						
	Trans-Sinapic	Methyl–4-OH-Benzoic	Protocate Chuic	Vanillic	Trans-Ferulic	P-Coumaric	Caffeic
*P. africana*	ND	515.54 ± 12.75	16.78 ± 0.64	ND	ND	13.38 ± 1.51	34.26 ± 0.61
*L. indica*	ND	244.8 ± 6.04	11.56 ± 0.5	ND	ND	11.33 ± 1.04	ND
*P. pinnata*	ND	347.4 ± 7.77	ND	ND	ND	ND	ND

## Data Availability

Data will be made available on request from the corresponding author and in the Supplementary Materials.

## Supplementary Materials

The following supporting information can be downloaded at https://www.mdpi.com/article/10.3390/antiox14060666/s1, Table S1: Tukey’s multiple comparison test for total phenolic and total flavonoid content of *Prunus africana*, *Leea indica* and *Paullinia pinnata* plant extract; Table S2: Tukey’s multiple comparison test for antioxidant activities of the plant extracts; Figures S1: Chromatogram of standard mix (external and internal standard) and Figures S2–S4: Chromatogram of sample extracts.
