# Supplementary material for: Phytochemical Analysis and Antioxidant Activities of Prunus africana Bark, Leea indica and Paullinia pinnata Leaf Extracts
Source: Antioxidants (Basel). 2025 May 30;14(6):666. doi: 10.3390/antiox14060666 (PMC12189430; doi:10.3390/antiox14060666)
Supplement: Supplementary file 1 [file antioxidants-14-00666-s001.zip › antioxidants-3637833-supplementary.pdf]

Supplementary materials

The List of Contents

No.	Contents	Page
1	Table S1. Tukey’s multiple comparison test for Total Phenolic and Total Flavonoid Content	S2
2	Table S2. Tukey’s multiple comparison test for antioxidant activities	S3
3	Figure S1: Chromatogram of standard mix (External and Internal)	S4
4	Figure S2: Chromatogram of *Prunus africana*	S4
5	Figure S3: Chromatogram of *Leea indica*	S5
6	Figure S4: Chromatogram of *Paullinia pinnata*	S5

Table S1: Comparison of TPC and TFC among the extracts. One-way ANOVA with error bars representing standard deviation differences, where *P < 0.05 and **P<0.01 were considered as significant, ns: not significant.

(a)

Total Phenolic Content (TPC)

Tukey's multiple comparisons test	Mean Diff.	95.00% CI of diff.	Below threshold?	Summary	Adjusted P Value
P. africana vs. *L. indica*	26.20	-9.563 to 61.96	No	ns	0.1408
P. africana vs. *P. pinnata*	75.20	39.44 to 111.0	Yes	**	0.0016
L. indica vs. *P. pinnata*	49.00	13.24 to 84.76	Yes	*	0.0134

(b)

Total Flavonoid Content (TFC)

Tukey's multiple comparisons test	Mean Diff.	95.00% CI of diff.	Below threshold?	Summary	Adjusted P Value
P. africana vs. *L. indica*	14.94	2.223 to 27.66	Yes	*	0.0263
P. africana vs. *P. pinnata*	23.50	10.78 to 36.22	Yes	**	0.0031
L. indica vs. *P. pinnata*	8.560	-4.157 to 21.28	No	ns	0.1776

Table S2: Comparison of antioxidant activities among the extracts. One-way ANOVA with error bars representing standard deviation differences, where*P < 0.05, **P < 0.01,***P < 0.001, and ****P<0.0001 were considered as significant, ns: not significant.

(a)

% of 2, 2-diphenyl-1-picrylhydrazyl (DPPH) scavenging activity

Tukey's multiple comparisons test	Mean Diff.	95.00% CI of diff.	Below threshold?	Summary	Adjusted P Value
P. africana vs. *L. indica*	4.700	-13.68 to 23.08	No	ns	0.7254
P. africana vs. *P. pinnata*	5.480	-12.90 to 23.86	No	ns	0.6515
L. indica vs. *P. pinnata*	0.7800	-17.60 to 19.16	No	ns	0.9907

(b)

IC_{50} for DPPH

Tukey's multiple comparisons test	Mean Diff.	95.00% CI of diff.	Below threshold?	Summary	Adjusted P Value
P. africana vs. *L. indica*	-22.60	-68.42 to 23.22	No	ns	0.3499
P. africana vs. *P. pinnata*	-46.60	-92.42 to -0.7800	Yes	*	0.0469
L. indica vs. *P. pinnata*	-24.00	-69.82 to 21.82	No	ns	0.3133

(c)

% of *(2,2-azino-bis (3-ethyl-benzothiazoline-6-sulfonic acid)) (ABTS)* scavenging activity

Tukey's multiple comparisons test	Mean Diff.	95.00% CI of diff.	Below threshold?	Summary	Adjusted P Value
P. africana vs. *L. indica*	5.800	-9.763 to 21.36	No	ns	0.5250
P. africana vs. *P. pinnata*	8.160	-7.403 to 23.72	No	ns	0.3127
L. indica vs. *P. pinnata*	2.360	-13.20 to 17.92	No	ns	0.8896

(d)

IC_{50} for ABTS

Tukey's multiple comparisons test	Mean Diff.	95.00% CI of diff.	Below threshold?	Summary	Adjusted P Value
P. africana vs. *L. indica*	-3.200	-33.03 to 26.63	No	ns	0.9426
P. africana vs. *P. pinnata*	-35.80	-65.63 to -5.966	Yes	*	0.0240
L. indica vs. *P. pinnata*	-32.60	-62.43 to -2.766	Yes	*	0.0354

(e)

Ferric ion reducing antioxidant power (FRAP)

Tukey's multiple comparisons test	Mean Diff.	95.00% CI of diff.	Below threshold?	Summary	Adjusted P Value
P. africana vs. *L. indica*	10.60	6.283 to 14.92	Yes	***	0.0007
P. africana vs. *P. pinnata*	17.70	13.38 to 22.02	Yes	****	<0.0001
L. indica vs. *P. pinnata*	7.100	2.783 to 11.42	Yes	**	0.0056

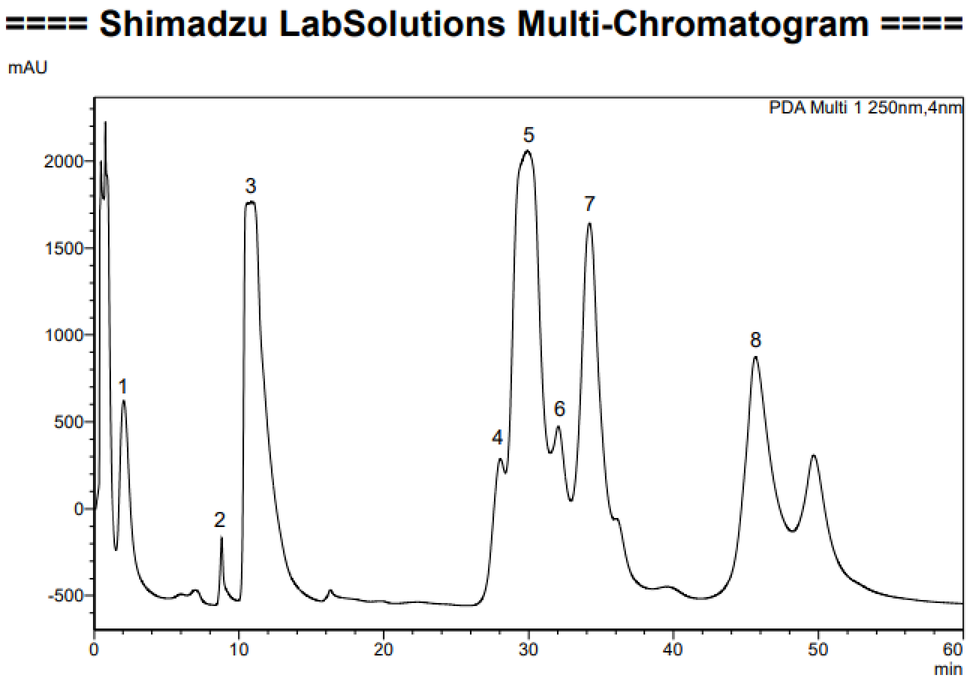

Figure S1: Chromatogram of standard mix (External and Internal)

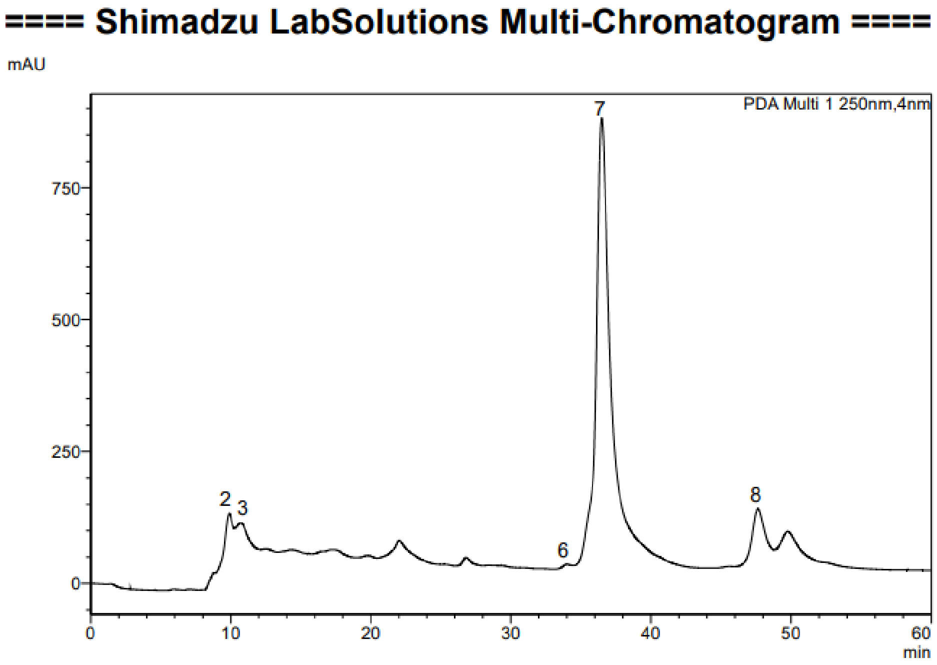

Figure S2: Chromatogram of *Prunus africana*

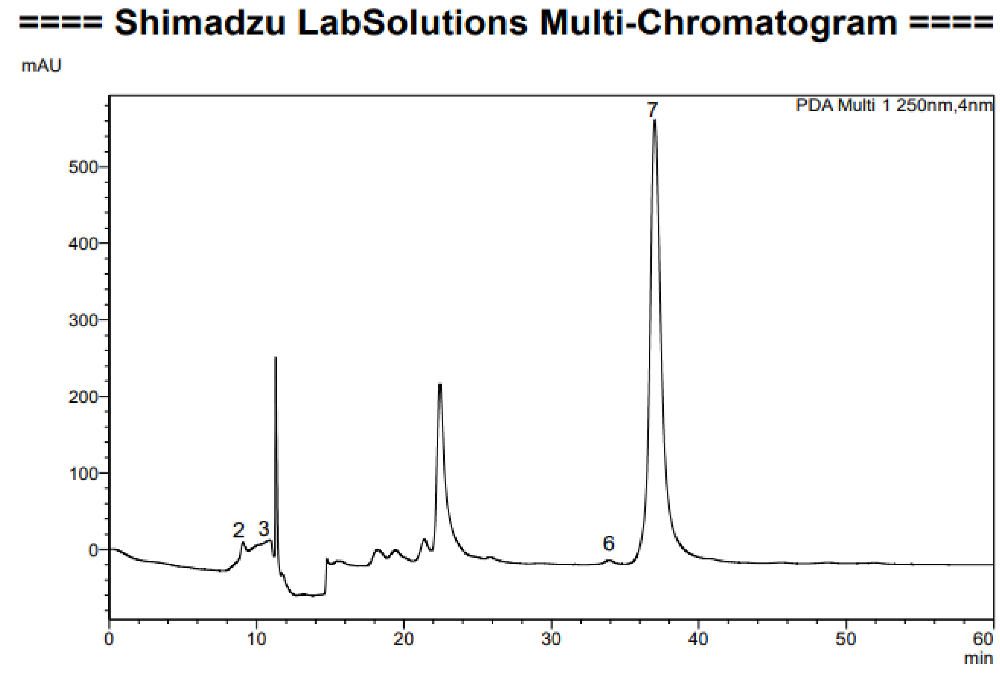

Figure S3: Chromatogram of *Leea indica*

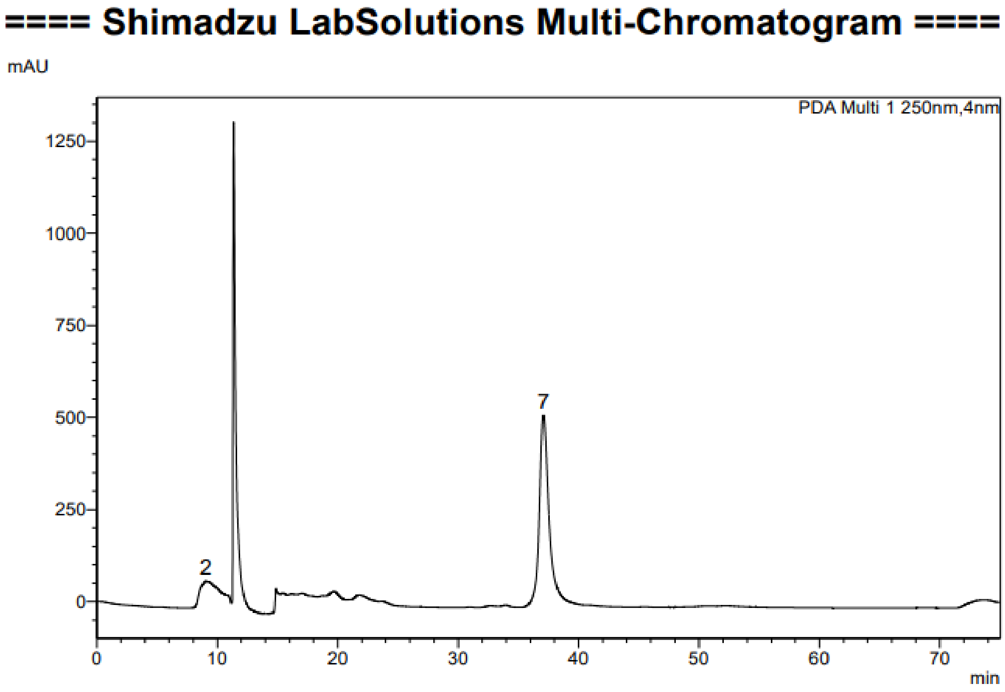

Figure S4: Chromatogram of *Paullinia pinnata*
